# Renal disorders in Autoimmune Polyendocrinopathy Candidiasis Ectodermal dystrophy (APECED): a systematic review

**DOI:** 10.1186/s12887-025-05458-2

**Published:** 2025-02-26

**Authors:** Mohammadreza Shafiei, Solale Hosseini, Soodeh Ghadimi, Mahbubeh Mirzaee, Mohammadamin Keikhah, Nastaran Ardalan, Masoumeh Mohkam, Mehrdad Tamiji, Mahnaz Jamee

**Affiliations:** 1 Student Research Committee, School of Medicine, Alborz University of Medical Sciences, Karaj, Iran; 2 School of Medicine, Azad University of Medical Sciences, Tehran, Iran; 3 Pediatric Nephrology Research Center, Research Institute for Children’s Health, Shahid Beheshti University of Medical Sciences, Tehran, Iran; 4 Department of Computer Science, Rice University, Houston, TX USA

**Keywords:** Autoimmune polyendocrinopathy, APECED, AIRE, Primary immunodeficiency, Inborn error of immunity, Nephrocalcinosis

## Abstract

**Background:**

Autoimmune polyendocrinopathy candidiasis ectodermal dystrophy (APECED), also known as autoimmune polyendocrine syndrome type I (APS-I) is an inborn error of immunity (IEI) with an immune dysregulation phenotype, mainly characterized by endocrine and non-endocrine manifestations including adrenal insufficiency, chronic mucocutaneous candidiasis, and ectodermal dystrophy. Renal disorders seem to be a significant morbidity in APECED patients, requiring further investigations.

**Methods:**

The literature search was conducted in PubMed, Web of Science, and Scopus databases using relevant keywords, and included articles were systematically reviewed regarding the clinical and immunological features. APECED patients with at least one nephrological complication were included.

**Results:**

Ninety-three APECED patients from 30 studies were identified. More than half of the patients (38,52%) presented nephrocalcinosis. The second and third most prevalent renal complications were tubulointerstitial nephritis (TIN) (23,31%), and hypertension (13,18%), respectively. Other less frequent renal disorders including renal tubular acidosis (RTA) glomerulonephritis were also reported among patients. Additionally urinary tract infections (UTI), were also common among cases (15,20.5%).

**Conclusions:**

Renal complications in APECED represent a significant issue that should be monitored and considered in managing these patients to preserve renal function and improve patients’ outcomes.

**Supplementary Information:**

The online version contains supplementary material available at 10.1186/s12887-025-05458-2.

## Introduction

 Autoimmune polyglandular syndrome type 1 (APS-1), also known as Autoimmune polyendocrinopathy-candidiasis-ectodermal dystrophy (APECED) is a systemic autoimmune disease that involves endocrine and non-endocrine organs [1]. Mutations in the Autoimmune Regulator (*AIRE*) gene are causative for APECED development [2]. The *AIRE* is located at chromosome position 21q22.3, comprising 14 coding exons that produces a transcription regulator of 545 amino acids, weighing 58 kDa [3]. *AIRE* plays a role in regulating the expression of tissue-specific antigens (TSAs), which are crucial for the process of negative selection by ensuring the presentation of the full range of self-antigens at the site of negative selection, leading to the removal of any self-reactive T cells, therefore, mutations in *AIRE* impair self-tolerance and increase autoreactive immune cells that target multiple tissues [4].

The clinical features of APECED are characterized by a triad of chronic mucocutaneous candidiasis (CMC), adrenal insufficiency (AI), and hypoparathyroidism (HP) [5]. Besides the classic triad, patients also suffer from other endocrine and non-endocrine manifestations. Based on recent estimations up to 10% of APECED patients experience at least one type of renal disorder [6]. Renal complications are caused by T-cell infiltration in renal tubules and the development of anti-proximal tubular and anti-collecting duct-specific autoantibodies in some APECED patients. Renal disorders range from mild renal impairment in patients with nephrocalcinosis to a rapidly progressive renal failure requiring kidney transplantation followed by tubulointerstitial nephritis (TIN) [1, 6, 7]. Early administration of immunosuppressive therapies to APECED patients with kidney complications led to the cessation of TIN progression in some patients, therefore, detection of renal involvement by monitoring renal function may improve patients’ outcomes [8]. Although more than 80% of patients with APECED have hypoparathyroidism, nephrocalcinosis is observed in some patients, suggesting that iatrogenic impaired calcium homeostasis may deteriorate patients’ condition [8, 9].

Regardless of previous investigations, estimations regarding the prevalence of renal complications in these patients are controversial, and the mechanism underlying the renal pathologies is not fully understood. In this study, we reviewed the characteristics of renal disorders and their correlation with genetic changes and polymorphisms and tried to better define the mechanism underlying renal pathologies of APECED patients. The result of this study may provide a better prognostic feature of involved individuals and impede inaccurate therapeutics that deteriorate renal function and patients’ prognosis.

## Methods

We adhered to the Preferred Reporting Items for systematic Reviews and Meta-Analyses (PRISMA) guidelines to ensure transparency and rigor in the study design, literature search, selection, and reporting processes.

### Search strategy

A comprehensive search was conducted using several databases including PubMed, and Web of Science up to January 1st, 2023, applying different terms: “Autoimmune polyendocrine syndrome type 1” OR “Polyglandular Autoimmune Syndrome type 1” OR “Autoimmune Polyglandular Syndrome type 1” OR “APS1” OR “Autoimmune polyendocrinopathy candidiasis ectodermal dystrophy” OR “APECED” OR “Autoimmune polyendocrinopathy type 1” OR “Autoimmune regulator gene” OR “AIRE gene” AND “Renal Insufficiency” OR “Renal Failure” OR “Kidney” OR “Renal Disease” OR “ESRD” OR “kidney diseases” OR “Hypercalcemia” AND “Hypercalciuria” OR “Hypertension” OR “Nephrotic Syndrome” OR “Nephritic Syndrome” OR “Glomerulonephritis” OR “Hydronephrosis” OR “Hyperoxaluria” OR “Nephritis” OR “Nephrocalcinosis” OR “Renal Tubular Transport” OR “Renal Tubular Acidosis” OR “Nephrolithiasis”. Patients reported in screened articles were further assessed for renal complications. We also identified potentially relevant literature by a manual search in references of all included studies.

### Study selection

The initial screening of articles was performed by two independent investigation teams, who reviewed titles/abstracts to include relevant studies according to the inclusion criteria.

The literature search was limited to articles written in the English language, and articles reporting at least one APECED patient with renal disorder were considered to be included. We excluded Studies using animal models, reviews and meta-analyses, and any types of secondary studies, conference abstracts, and articles in other languages. Further screening was performed by reviewing full texts.

### Data extraction

Two independent investigation teams collected the following data: publication year and author, age, gender, country, gene defect, the number of participants, and renal and non-renal disorders and other abnormalities. Duplicate data was removed, and a third researcher resolved discrepancies between the two investigators.

## Results

### Study characteristics

**Fig. 1:**
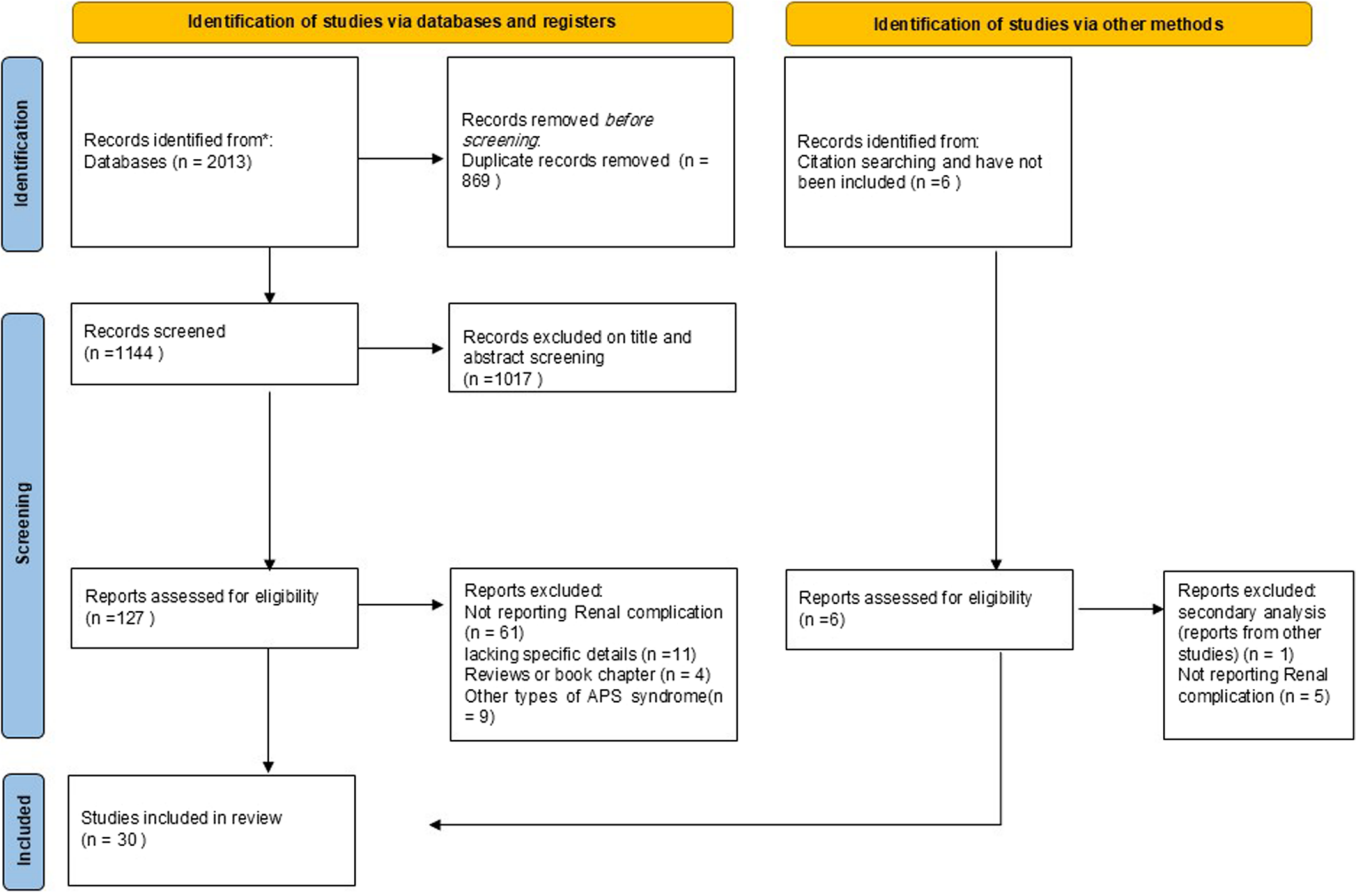
PRISMA diagram summarizing the selection of eligible studies based on the PRISMA guideline

After the removal of duplicates, a total of 1144 articles were assessed based on title and abstract. Of these, 1017 articles were excluded; 127 remaining articles were further reviewed by reviewing the full text. Data was extracted from 30 articles fulfilling the inclusion criteria. A total of 93 patients were finally enrolled in our study. The Prisma diagram is shown in Fig. 1. The list of extracted data including articles and patients is shown in Table 1.

**Table 1:** Summary of renal disorders in patients with APECED

Pt No.	Country	Gene Defect	Sex	Age at study	AOO	AOD	Endocrinopathy	Ectodermal dystrophy
*		**19 patients (out of 27) with nephrocalcinosis (Rows 1–19)** 92% of the adults and 54% of children in the cohort had evidence of nephrocalcinosis. An average rhPTH1-34 dose of 9.6 ± 1.4 µg was administered by subcutaneous injection Mean calcium levels remained stable and ranged from 2.06 to 2.17 mmol/L with minimal fluctuation.						
1	USA	-	F	4	2	-	HP	-
2	USA	-	M	17	8	17	HP	-
3	USA	-	F	9	3	-	HP	-
4	USA	-	F	15	9	10	HP	-
5	USA	-	M	16	13	14	HP	-
6	USA	-	F	17	4	10	HP	-
7	USA	-	M	16	6	15	HP	-
8	USA	-	M	41	9	9	HP	-
9	USA	-	F	37	5	15	HP	-
10	USA	-	F	35	4	-	HP	-
11	USA	-	F	35	1	10	HP	-
12	USA	-	F	28	9	9	HP	-
13	USA	-	F	42	11	18	HP	-
14	USA	-	F	42	7	-	HP	-
15	USA	-	F	21	3	11	HP	-
16	USA	-	F	35	4	4	HP	-
17	USA	-	F	55	3	13	HP	-
18	USA	-	F	54	0.5	10	HP	-
19	USA	-	M	53	6	14	HP	-
20	Turkey	AIRE: a homozygous frameshift mutation (p.Asp70fs, c.208_209insCAGG) in exon 2, in AIRE gene	M	13	5	10	Autoimmune Diabetes, PAI	No
21	Portugal	Hemozygosity for the pathogenic variant c.1103dup p. (Leu370Alafs*2), a frameshift mutation resulting in the introduction of a premature stop codon	M	50	-	7	HP, PAI	Bilateral cataracts and recurrent keratitis, transient alopecia, onychomycosis,
22	North or south America	c.967_979del13 mutation in homozygosity	2 patients Gender not available	-	-	12.1	HP	Enamel hypoplasia
23	Austria	com- pound heterozygous mutations in the AIRE-gene, c.931del and c.967_979de	F	5	-	-	HP	No
24	Ethnicity: Caucasian Country: USA	Allele 1: c.967_979del13 Allele 2: c.1249_1250insC	F	19	11	-	HP, PAI	ENAMEL HYPOPLASIA,
25	Ethnicity: Caucasian Country: USA	Allele 1: c.967_979del13 Allele 2: c.769 C > T	F	10	-	-	HP, PAI	ENAMEL HYPOPLASIA,
26	Italy	a heterozygous R203X mutation, in exon 5 in the SAND domain (amino acid 181–280)	F	7	-	-	chronic thyroiditis, subclinical hypothyroidism	Alopecia
27	Finland	R257X/R257X	1	-	2	-	Autoimmune thyroiditis, PAI, HP, hypogonadism	Enamel hypoplasia, alopecia
28	Brazil	-	1	28	-	-	HP	Dental enamel and nail dysplasia, corneal keratopathy
29	Ireland	c.967-979del at exon 8 p.L323_L327 > SfsX51	1	12	-	-	HP, PAI	Superior Corneal Vascularization, Reduced Tears
30	Brazil	c.[560 C > G]; [560 C > G] p.[(Ser187*)]; [(Ser187*)]	1	-	-	-	HP, PAI, hypogonadism	Dental enamel hypoplasia, nail dystrophy, keratopathy
31	Italy	P539L	1	7	-	-	HP, PAI	Enamel hypoplasia l
32	Italy	1058delT and 1058delT	0	23	-	-	HP	Alopecia, Enamel hypoplasia
33	Italy	W78R and W78R	0	42	-	-	PAI, HP	Alopecia, Enamel hypoplasia
34	Italy	W78R and W78R	1	37	-	-	HP, hypogonadism	Alopecia, Enamel hypoplasia
35	Lebanon	c. (132 + 1 133-1) (463 + 1_464-1) del p. Glu45Alafs*3	0	15	-	-	HP	Nail Dystrophy, Cataract
36	Italy	R203X	1	28	-	-	HP, PAI, POF	-
37	Canada	-	1	29	-	13	Thyroiditis, hypothyroidism, hypogonadism	-
38	Iran	-	1	12	-	-	HP, IDDM	Alopecia universalis
39	Iran	-	0	15	-	-	Thyroiditis, HP, hypothyroidism	Alopecia universalis
40	Iran	c.1236 1237insGCCG p.Leu414GlyfsX12	1	17	-	-	PAI, HP, POI	No
41	Iran	c.93 94insT p.Leu32SerfsX3	1	12	-	-	Thyroiditis, PAI, HP, hypothyroidism	Alopecia universalis
42	Iran	c.205 208dupCAGG p.Asp70AlafsX148	0	35	-	-	PAI, HP	No
43	Turkey	-	1	36	-	-	PAI, HP, ovarian failure	No
44	Finland	-	1	41	-	-	PAI, HP, Ovarian Atrophy	Alopecia Universalis
45	Japan	-	-	-	-	-	Autoimmune thyroiditis, IDDM	-
46	Iran	large homozygous deletion encompassing exons 2– 4 (g.424_2157del1734) in the AIRE gene	1	23	-	-	Thyroiditis, HP, PAI, hypothyroidism	Alopecia, keratitis punctata
47	Netherlands	c.967_979del13+ [(?_68)_(1567-14_? )del] p.[Cys322fs]+(0?)	0	15	-	-	PAI	No
48	Saudi Arabia	c.845 846insC/ 4q 33 deletion p.Leu283SerfsX6	0	-	-	-	PAI, HP	Nail dystrophy
49	China	c.206 A > C p.Q69P	0	21	-	-	PAI, HP	Enamel dysplasia
*		Kidney Transplantations And Clinical Characteristics In Patients With TIN Due To APECED (Aoos Are The Ages At Kidney Transplantation) 6 Rows Below:						
50	Finland	c.769 C > T/ c.769 C > T	F	-	15–20	-	POI, HP, PAI	Iridocyclitis
51	Finland	c.967–979del13bp/x	M	-	10–15	-	Hypothyroidism, PAI, growth hormone deficiency	-
52	Finland	c.769 C > T/c.769 C > T	F	-	20–25	-	Hypothyroidism, HP, PAI, POI	Alopecia
53	Finland	c.769 C > T/c.769 C > T	F	-	25–30	-	PAI, Hypothyroidism, HP, POI	Glaucoma
54	Finland	c.769 C > T/c.769 C > T	F	-	First KT:220–25 Second KT: 50–55	-	PAI, HP	Alopecia
55	Finland	c.769 C > T/c.769 C > T	F	-	First KT: 45–50 Second KT: 50–55	-	HP, PAI, POI, DM	-
*		*N* = 30 Patients with Confirmed APECED Syndrome (Percentages Belong to All 30 Patients) *N* = 14 Patients with Confirmed APECED Syndrome Who Had Experienced At Least One Kidney/Urinary Tract-Related Symptom During Lifetime (Row 56) Among The Five Patients with Diminished GFR, Three Women(*N* = 3) (3/30, 10%) Had A Chronic Renal Failure Related To TIN, Confirmed by A Kidney Biopsy (Rows 57–59)						
56	Finland	-	Male :10 Female :20(of all APECED patients available)	Mean (± SD): 40 ± 15.5 Range: 7–68	Mean: 4 ± 3.6	Mean: 8 ± 4.8	HP (83%), PAI (76%), Hypogonadism (48%), Hypothyroidism (33%), DM-1(16%), Growth hormone deficiency (6%),	Alopecia areata/hair loss (50%), Keratitis (23%)
57	Finland	the Finn major AIRE mutation (R257X×2)	F	48	-	20	-	-
58	Finland	the Finn major AIRE mutation (R257X×2)	F	27	-	16	-	-
59	Finland	the Finn major AIRE mutation (R257X×2)	F	12	-	9	-	-
60	Ethnicity: Caucasian Country: Poland	-	F	14	9	-	HP, subclinical Hashimoto’s disease	Dental enamel hypoplasia

Pt No.	CMC	Other abnormalities	Renal disorders	Ca replacement therapy (Type)	Other treatments	Outcome	Reference	
*	**19 patients (out of 27) with nephrocalcinosis (Rows 1-19)** 92% of the adults and 54% of children in the cohort had evidence of nephrocalcinosis. An average rhPTH1-34 dose of 9.6 ± 1.4 µg was administered by subcutaneous injection Mean calcium levels remained stable and ranged from 2.06 to 2.17 mmol/L with minimal fluctuation.						[10]	
1	-	-	Nephrocalcinosis	25-OH-Vit D, rhPTH 1-34	-	-	[10]	
2	-	-	Nephrocalcinosis	25-OH-Vit D, rhPTH 1-34	-	-	[10]	
3	-	-	Nephrocalcinosis	25-OH-Vit D, rhPTH 1-34	-	-	[10]	
4	-	-	Nephrocalcinosis	25-OH-Vit D, rhPTH 1-34	-	-	[10]	
5	-	-	Nephrocalcinosis	25-OH-Vit D, rhPTH 1-34	-	-	[10]	
6	-	-	Severe Nephrocalcinosis	25-OH-Vit D, rhPTH 1-34	-	-	[10]	
7	-	-	Nephrocalcinosis	25-OH-Vit D, rhPTH 1-34	-	-	[10]	
8	-	-	Nephrocalcinosis	25-OH-Vit D, rhPTH 1-34	-	-	[10]	
9	-	-	Nephrocalcinosis	25-OH-Vit D, rhPTH 1-34	-	-	[10]	
10	-	-	Severe Nephrocalcinosis	25-OH-Vit D, rhPTH 1-34	-	-	[10]	
11	-	-	Severe Nephrocalcinosis	25-OH-Vit D, rhPTH 1-34	-	-	[10]	
12	-	-	Nephrocalcinosis	25-OH-Vit D, rhPTH 1-34	-	-	[10]	
13	-	-	Nephrocalcinosis	25-OH-Vit D, rhPTH 1-34	-	-	[10]	
14	-	-	Nephrocalcinosis	25-OH-Vit D, rhPTH 1-34	-	-	[10]	
15	-	-	Severe Nephrocalcinosis	25-OH-Vit D, rhPTH 1-34	-	-	[10]	
16	-	-	Moderate Nephrocalcinosis	25-OH-Vit D, rhPTH 1-34	-	-	[10]	
17	-	-	Moderate Nephrocalcinosis	25-OH-Vit D, rhPTH 1-34	-	-	[10]	
18	-	-	Moderate Nephrocalcinosis	25-OH-Vit D, rhPTH 1-34	-	-	[10]	
19	-	-	Severe Nephrocalcinosis	25-OH-Vit D, rhPTH 1-34	-	-	[10]	
20	Yes	JIA, recurrent oral candidiasis, chronic diarrhea, Generalized hyperpigmentation, necrotizing pneumonia and lung abscess, functional hyposplenism, Eosinophilic ileitis and focal active colitis, Autoimmune enteropathy	TIN	-	Steroids, Antibacterial therapy and prophylaxis, antifungal, IVIG, insulin, sodium bicarbonate therapy, azathioprine, and sirolimus	Alive	[11]	
21	Yes	generalized tonic-clonic seizures, ocular disorders, chronic, gastritis, extraskeletal calcifications, cholelithiasis, weakness, musculoskeletal pain, fatigue, nausea, anorexia, weight loss, dyspepsia, dysphagia (even under esomeprazole), constipation, flatulence, occasional paresthesia in the extremities, mucocutaneous hyperpigmentation, a Bethesda IV thyroid nodule	Nephrolithiasis	elemental calcium (calcium lactate gluconate and calcium carbonate), calcitriol	selective laser trabeculoplasty, topical minoxidil, hemithyroidectomy, cholecystectomy, Antifungal therapy, hormonal replacement: hydrocortisone, mineralocorticoid replacement, oral fluconazole topical treatment with ciclopirox olamine for onychomycosis, levothyroxine, esomeprazole	Alive	[12]	
22	yes	unattributable	Tubulointerstitial Nephritis	-	-	-	[13]	
23	No	mild chronic diarrhea since infancy, generalized tonic-clonic seizures	Mild Hypercalciuria (To Date, Not Seen AnySigns of Nephrocalcinosis or Nephrolithiasis)	high-dose therapy with oral calcium,alfacalcidol , recombi-nant rhPTH	-	Alive	[14]	
24	No	chronic colitis, Ovarian failure, pneumonitis, hypertension	Tubulointerstitial Nephritis	-	Azathioprine, MPS	-	[15]	
25	Yes	gastritis, pneumonitis,alopecia, hypothyroidism, Sjogren’s-like syndrome	Tubulointerstitial Nephritis	-	MMF, Azathioprine	-	[15]	
26	-	episodic weakness, chronic vaginal mycosis, stomach pains	LKM Ab Positive with No Other Renal Dysfunction.	-	-	-	[16]	
27	Yes	tongue SCC in situ	Tubulointerstitial Nephritis	-	SCC radical resection, Photodynamic therapy	-	[17]	
28	Yes	malabsorption, bronchiectasis, pernicious anemia, autoimmune thrombopenia, autoimmune gastrointestinal disease)	Tubulointerstitial Nephritis	-	-	-	[18]	
29	Yes	Epilepsy	Recurrent UTI	-	-	-	[19]	
30	Yes	pernicious anemia, autoimmune thrombopenia, intestinal dysfunction	Nephropathy (no further details)	-	-	-	[20]	
31	Yes	-	Nephrocalcinosis, Multicystic Left Kidney	-	-	-	[21]	
32	Yes	malabsorption	Nephrocalcinosis	-	-	-	[21]	
33	Yes	Tympanic calcifications, Nephrocalcinosis, esophageal carcinoma, optic nerve atrophy, Atrophic gastritis, Cataract, chorioretinitis	Nephrocalcinosis	-	-	-	[21]	
34	Yes	Sjogren's syndrome Atrophic gastritis, Cataract	Nephrocalcinosis	-	-	-	[21]	
35	Yes	Hepatitis autoimmune hepatitis	Nephrocalcinosis	-	Azathioprine	-	[22]	
36	No	Epilepsy	Nephrocalcinosis	-	-	-	[23]	
37	-	arthritis	Nephrocalcinosis	-	-	-	[24]	
38	Yes	vit B12 deficiency	Nephrocalcinosis	-	-	-	[25]	
39	Yes	celiac disease, iridiocyclitis	Nephrocalcinosis	-	-	-	[25]	
40	Yes	-	Nephrocalcinosis	-	-	-	[25]	
41	Yes	-	Nephrocalcinosis	-	-	-	[25]	
42	Yes	-	Nephrocalcinosis	-	-	-	[25]	
43	No	Asplenism, pernicious anemia, autoimmune thrombopenia, corneal vascularization,corneal scarring	Nephrolithiasis	-	prednisone, prednisolone acetate	-	[26]	
44	Yes	SCC,	TIN	-	-	-	[27]	
45	-	Intestinal dysfunction, autoimmune hepatitis	Glomerulonephritis	-	-	-	[28]	
46	Yes	generalized seizures, severe cerebral atrophy and an arachnoid cyst of the posterior fossa, myocardiopathy, pernicious anemia, autoimmune thrombopenia, sicca syndrome, chronic diarrhea, exocrine pancreatic insufficiency,	ESRD, TIN	-	-	-	[29]	
47	Yes	chronic/tension headache	Tubulointerstitial Nephritis	-	-	-	[30]	
48	Yes	craniofacial features, FTT, Diffuse cerebellar atrophy, profound psychomotor retardation, recurrent vomiting	Glomerulonephritis	-	-	-	[31]	
49	Yes	fatigue and hypercalcemic tetany, vertigo, chronic intestinal dysfunction, binocular cataract	Glomerulonephritis	-	Steroid therapy	-	[32]	
*	Kidney Transplantations And Clinical Characteristics In Patients With Tubulointerstitial Nephritis Due To APECED (Aoos Are The Ages At Kidney Transplantation) 6 Rows Below:							
50	Yes	rash with fever Infections requiring hospitalization: Mastoiditis; Pyelonephritis *Proteus mirabilis*	Tubulointerstitial Nephritis	-	immunosuppressive treatment: Basiliximab + CsA + MMF + steroids anti-CD20 therapy	At the age of 19 y alive with functioning graft (mGFR, 39 mL/min)	[33]	
51	Yes	rash with fever, AIHA, Hepatitis, exocrine pancreas insufficiency	Tubulointerstitial Nephritis	-	immunosuppressive treatment: CsA+ Aza+ steroids	Deceased at 19 y with a functioning graft	[33]	
52	Yes	Hyposplenia Epithelial carcinoma of tongue, Infections requiring hospitalization: Pneumocystis carinii, campylobacter jejuni	Tubulointerstitial Nephritis	-	immunosuppressive treatment: CsA + Aza+ steroids	At the age of 54 y alive with functioning graft (eGFR, 71 mL/min)	[33]	
53		Infections requiring hospitalization: *;* Escherichia coli pyelonephritis; Cholecystitis, peritonitis and E coli septicemia.	Tubulointerstitial Nephritis	-	immunosuppressive treatment: CsA+ MMF + steroids	At the age of 33 y alive with functioning graft (eGFR, 36 mL/min)	[33]	
54	Yes	atrophic gastritis Infections requiring hospitalization: Enterococcus faecalis pyelonephritis; Staphylococcus aureus septicemia	Tubulointerstitial Nephritis	-	immunosuppressive treatment: CsA + Aza+ steroids After second KT: Tac + MMF + steroids	Return to dialysis in 2013 Deceased at 58 y with a functioning graft (eGFR, 25 mL/min)	[33]	
55	Yes	-	Tubulointerstitial Nephritis	-	immunosuppressive treatment: CsA + MMF + steroids After second KT: Basiliximab + Tac + MMF + steroids	Graft removed 1 y post-KT Deceased at 56 y with a functioning graft (eGFR, 70 mL/min)	[33]	
*	N=30 Patients with Confirmed APECED Syndrome (Percentages Belong to All 30 Patients) N=14 Patients with Confirmed APECED Syndrome Who Had Experienced At Least One Kidney/Urinary Tract-Related Symptom During Lifetime (Row 58) Among The Five Patients with Diminished GFR, Three Women(N=3) (3/30, 10%) Had A Chronic Renal Failure Related To TIN, Confirmed by A Kidney Biopsy (Rows 59-61)							
56	Yes, 96%	11 patients with HTN, Asplenia (30%), Vitiligo (23%), Pernicious anemia (6%), Autoimmune hepatitis (6%),	14 UTI, 2 Nephrolithiasis, 2 Nephrocalcinosis, 2 RTA, 3 TIN, 2 Diabetic Nephropathy	-	-	-	[8]	
57	-	-	A Chronic Renal Failure Related To TIN (A Renal Transplantation (33 Y/O))	-	cyclosporine	Alive	[8]	
58	-	HTN	TIN With Nephrocalcinosis	-	-	Alive, currently on hemodialysis	[8]	
59	-	-	TIN, RTAType 1	-	Supportive treatment by sodium bicarbonate, Mycophenolatemofetil (MMF), rituximab	Alive, progressiveworsening of kidney function	[8]	
60	Yes, Oral candidiasis	syncope andseizure,	Nephrocalcinosis	calcium supplementation and 1-α-hydroxycholecalciferol administration	-	-	[34, 35]	

F female, M male, AOO age of onset, AOD age of diagnosis, CMC chronic mucocutaneous candidiasis, Ca calcium, MPS mycophenolate sodium, rhPTH recombinant human parathyroid hormone, Vit D vitamin D, JIA juvenile idiopathic arthritis, TIN tubulointerstitial nephritis, RTA renal tubular acidosis, ESRD end-stage renal disease, HTN hypertension, HP hypoparathyroidism, POI primary ovarian insufficiency, PAI primary adrenal insufficiency, DM diabetes mellitus, UTI urinary tract infection, LKM Ab Anti-liver-kidney microsomal antibody, MMF Mycophenolate Mofetil, KT kidney transplant

### Case characteristics

A total of 73 patients were evaluated (42 males, 17 females, and 14 patients of unknown sex). At the time of the study, 10 patients were alive (live status only for 13 of a total of 73 patients were available). The median age of patients at the diagnosis of the disease, and at the study was 9.7, and 30.7 years, respectively (from available cases). Most patients were from Finland (41,44%), the United States (23,25%), Italy (6,6.5%), Iran (6,6.5%), Brazil (3,32%), and Turkey (2,21%). Other cases were reported in diverse countries including Portugal, Austria, Ireland, Lebanon, Canada, Japan, China, Netherlands, Saudi Arabia, and Poland. Only the genetic data of 33 patients were available. For thirty-five patients, at least one type of gene defect was described. *AIRE* mutations c.967-979del13bp (p. L323Sfs*51) in 7 patients (22%) was the most repeated mutation, followed by c.769 C > T (p.R257*) mutation in 6 patients (19%) and 11 Alleles.

### Clinical features

The classical triad of APECED comprises CMC, HP, and adrenal insufficiency (AI). Of these, HP was the most prevalent with presentation in 90% (53 of 59) of patients, pursued by CMC (86%, 30 of 35) and AI (44%, 26 of 59).

Most of the patients (59, 80.8%) had at least one type of endocrinopathy, 16 patients (21.9%) had two types of endocrinopathy, 9 patients (12.3%) had three types of endocrinopathy, and 6 patients (8.2%) had four types of endocrinopathy.

Hypoparathyroidism (90%, 53 of 59 reported) was the most common complication among endocrinopathies, followed by primary adrenal insufficiency (PAI) (44%, 26 of 59), hypogonadism (8.5%, 5 of 59), hypothyroidism (27%, 16 of 59), primary ovarian insufficiency (8.5%, 5 of 59). Diabetes mellitus (3%, 2 of 59), subclinical thyroiditis (3%, 2 of 59), growth hormone deficiency (3%, 2 of 59), single occurrence of chronic thyroiditis, and ovarian atrophy.

Ectodermal dystrophy occurred as the most prevalent non-triad manifestation in APECED patients 37% (27 of 73). In addition, two patients showed more than two types of ectodermal dystrophy. Among patients with ectodermal dystrophy, the most prevalent form of ectodermal dystrophy was alopecia (18, 66.6%), with enamel hypoplasia (10, 37%), cataract (6, 22.2%), keratitis (2, 7.4%) and nail dystrophy (3,11.1%) following behind. Other ectodermal dystrophies such as onychomycosis, nail dysplasia, corneal keratopathy, superior corneal vascularization, tear reduction, and glaucoma were among other complications.

Besides routine clinical manifestations in APECED patients including autoimmune endocrinopathy, mucocutaneous candidiasis, and ectodermal dystrophy, about 44% of patients (32 of 73) showed a wide spectrum of other abnormalities, including more than seventy different diseases or symptoms (Table 1).

### The spectrum of renal disorders

APECED patients suffer from various types of renal disorders including nephrocalcinosis, hypertension, end-stage renal disease (ESRD), renal tubular acidosis (RTA), diabetic nephropathy, multicystic kidney disease, vasculitis, glomerulonephritis, and hypercalciuria. In this study, more than half of patients (38,52%) presented nephrocalcinosis which was severe in 5 cases and moderate in 3 cases. Besides nephrocalcinosis, four patients had nephrolithiasis, and one patient had mild hypercalciuria. The second most prevalent renal complication among patients was TIN (23, 31.5%); one of the TIN cases had chronic renal failure related to TIN. Hypertension was the third most prevalent (13,18%) renovascular involvement among patients. Autoimmune renal disorders were also prevalent among patients; three patients (4%) had glomerulonephritis (one of them had ANCA-mediated crescentic glomerulonephritis), and three cases (4%) had renal tubular acidosis (RTA). One case had positive LKM antibody but not any other diseases. In addition to the renal disorders, urinary tract complications including urinary tract infections (UTI), were common among cases (15,20.5%).

At the time of this study, in patients with available life status, three patients were alive (at the ages of 19, 33, and 54) with a functioning kidney graft, and three patients deceased at the ages of 19, 56, and 58 with a functioning graft. Two patients were under dialysis and one patient had a progressive worsening of kidney function. The graft was removed one year after kidney transplantation for one patient.

### Therapeutic strategies

Multiple therapeutic strategies were used to ameliorate patients’ symptoms and outcomes. About 32% of all patients had received one type of Ca/vit D/PTH replacement therapy. Of 24 cases who received Ca/vit D/PTH replacement therapy, 19 were under treatment with 25-OH-rhpTH1-34, 3 patients were under treatment with elemental calcium and calcitriol, one case was under treatment with high dose therapy with oral calcium, alfacalcidol (analogue of vitamin D), and recombinant PTH. Another case was under treatment with calcium supplementation and 1-alpha hydroxycholecalciferol administration.

Patients also received diverse medications which might cause or affect renal complications including sodium bicarbonate, intravenous immunoglobulin (IVIG), rituximab, and prophylactic antibacterial or antifungal therapy. Twenty-one patients (29%) were under treatment with immunosuppressive agents, of these, six patients received Mycophenolate mofetil (MMF), two patients were under treatment with steroids, three patients were under treatment with the combination of cyclosporine A (CSA), azathioprine, and steroids, three patients were under treatment with azathioprine, two patients were under treatment with CSA + MMF + steroids, and single cases were under treatment with basiliximab + CSA + MMF + steroids, basiliximab + MMF + Tacrolimus + steroids, Tacrolimus + MMF + steroids, cyclosporine, and azathioprine.

Other therapeutic and prophylactic agents used to improve the conditions and outcomes of patients are presented in Table 1.

## Discussion

Autoimmune polyendocrinopathy candidiasis ectodermal dystrophy (APECED) or Autoimmune Polyendocrine Syndrome type 1 (APS type 1) is a rare genetic syndrome diagnosed typically in childhood and late adolescence [9]. Renal disorders have recently been reported in 6.3–10% of APECED patients [36]. APECED patients suffer from a variety of renal diseases, which include renal tubular acidosis, nephrocalcinosis, tubulointerstitial nephritis, acute tubular necrosis, cystitis, pyelonephritis, nephrolithiasis, persistent proteinuria, diabetic nephropathy, microalbuminuria, and in later stages chronic renal failure. In the present study, we evaluated the incidence of renal complications and their association with genetic variations.

The most common mutation in APECED patients with renal disorders is consistent with previous reports and is the same as other APECED patients (35.8% of APECED patients had c.967-979del13bp (p. L323Sfs*51) mutation, which is also the most common mutation in included patients in our study). A study showed that while 3% of mutation-positive probands had tubulointerstitial nephritis, none of mutation-negative probands showed this renal complication, suggesting a potential high impact of genetic mutations in this renal involvement [37]. Several unique mutations have been reported in the included studies. However, it is not fully understood how genetic mutations could affect renal complications, and future studies should be carried out to find the associations between mutations and renal complications in APECED.

In this review, the most common cause of renal involvement in APECED patients is nephrocalcinosis. The prevalence of nephrocalcinosis in this review was 52%.

Several clinical conditions may induce nephrocalcinosis during the course of the disease, such as vitamin D replacement, congenital hypothyroidism, inherited tubulopathies, and RTA; hypoparathyroidism-induced hypercalciuria seems to be the most common cause as the fractional excretion of calcium increases and the tubular absorption of calcium decreases due to the lack of PTH. Nephrocalcinosis occurs through hypercalciuria with hypercalcemia or hypercalciuria with no hypercalcemia [38]. A study by Laakso and colleagues showed that APECED patients suffered from lower serum calcium, higher serum phosphate, and higher urine calcium secretion [39]. Therefore, hypercalciuria-induced without hypercalcemia nephrocalcinosis in APECED patients is probably the most among these patients. Based on clinical and laboratory findings in APECED patients; to elucidate the underlying mechanism of nephrocalcinosis in APCED patients, we suggested two theories. First, mutations in *calcium-sensing receptors (CASR)* can suppress PTH levels and exert a hypercalciuric hypocalcemia condition. Circulating autoantibodies against CASR in APECED patients, particularly activating antibodies, consolidate this theory [40, 41]. Second, vitamin D therapy may cause iatrogenic hypercalciuria at the initiation of treatment, therefore ongoing assessment of nephrocalcinosis is warranted [42]. It is suggested that calcium should be maintained just below or within the lower normal range to prevent nephrocalcinosis, nephrolithiasis, and renal failure [43]. Novel therapies such as recombinant PTH could maintain serum calcium levels without requiring intravenous calcium injection, which may exacerbate nephrocalcinosis [10].

In this study, the second form of renal involvement in patients with APECED was TIN, with a 32% prevalence. TIN is a severe complication of APECED that might ultimately lead to renal failure. Its prevalence in Finnish APECED patients is about 10% [8]. Severe pediatric cases of renal failure associated with TIN result in dialysis and kidney transplantation [1, 44]. In APECED, the only clinical sign of TIN may be impaired renal function without any urinalysis abnormalities [8]. Recently, the role of circulating circulating metabolites and antibodies and their prognostic value in nephritis have been shown [45]. Circulating antibodies against the proximal and distal parts of the nephron are present in TIN patients, with kidney biopsies showing lymphocyte infiltration and complement components deposition. Autoantibodies against proximal renal tubules in some cases indicate involvement of autoimmune pathogenic mechanisms in the development of renal complications in some patients [8]. The treatment of TIN is not clearly defined, but early initiation of immunosuppressive therapy such as MMF reduces the formation of fibrosis in the kidney via reducing circulating antibodies and diminishing T cell activity [29, 46].

The mechanism underlying kidney injuries could vary from metabolites dysfunctions, mitochondrial dysfunction, impaired autophagy, and activation of cell death pathways to immunologic processes such as infiltration of inflammatory cells, activation of inflammatory cytokines, and accumulation of macrophages [47, 48]. As mentioned above, renal complications are caused by T-cell infiltration in renal tubules and the development of anti-proximal tubular and anti-collecting duct-specific autoantibodies in the case of APECED.

The role of autoantibodies in the pathogenesis of kidney disorders in APECED is a matter of controversy. While some studies suggest that autoantibodies can rarely cause renal injury, the presence of these autoantibodies in some cases advocates the involvement of autoimmune pathogenic mechanisms in the development of renal complications in some patients, even though the main immunopathogenesis of APECED is the manipulated T-cell tolerance [7]. Hence, this could be a potential underlying mechanism of kidney involvement in APECED. Nevertheless, there are several types of autoantibodies against tissues, interferons, and interleukins in APECED patients. It is of not to say there were several autoantibodies in patients in this study such as anti tubular epithelium that were not reported in the previous comprehensive review of APECED patients [9]. These specific autoantibodies could also be used to predict renal involvement, as a previous study suggested that the measurement of autoantibodies produced against the cytokines could be used for early diagnosis of APECED [49].

No anti-proximal tubular and anti-collecting duct-specific autoantibodies were reported in the mentioned review. Therefore, other antibodies such as liver-kidney microsomal (LKM) or Anti-nuclear Ab (ANA), could also participate in the renal disorders’ development, particularly TIN.

The prevalence of other renal complications is inconsistent between our findings and previous reports. Hypertension was reported in 18% of included patients in this study, whereas the prevalence of hypertension was 36% in a Finnish cohort [8]. Since proteinuria and hypertension diminish renal function, early detection and management of these conditions seem to be necessary to preserve renal function in these patients, and screening for hypertension, proteinuria, and other renal function indicators should be considered in the examination of these patients. Since the onset of hypertension occurs during childhood in these patients, secondary screening tests such as plasma aldosteronism-to-renin ratio (ARR) test should be done to rule out other causes of hypertension and avoid cardiovascular damage [50]. Our study revealed that the occurrence of RTA was found to be 4% among renal complications in APECED, however, a previous Finnish study reported a higher prevalence of RTA at 6% among 30 cases [8]. In the context of APECED, autoantibodies predominantly target specific intracellular enzymes within organs. These autoantibodies are typically not pathogenic but rather signify ongoing T-cell activity within the affected tissue. A plausible hypothesis suggests a potential association between the presence of ductal cell antibodies and the development of distal renal tubular acidosis (type 1) observed in a subset of patients [51]. This rationale could be extended to APECED, even though future investigations are required to clarify the underlying mechanisms.

It is noteworthy to declare that while in the study by Sharifinejad and colleagues, 92% of patients were alive, our study showed that about 77% were alive (of reported cases) at the time of the study. This might show higher mortality in patients with kidney involvement, even though there is a lack of reported data on the living status of patients with kidney disorders is an obstacle to accurate estimation. Most of the cases included in our study were from Finland followed by the United States, Italy, Iran, Brazil, and Turkey. Whereas the Sharifinejad and colleagues, Italian cases were the second reported cases (followed by finish people), which suggest lower renal manifestations in Italian patients. The frequency of *AIRE* mutations was as same as the previous reports. The clinical presentation of APECED patients with kidney involvement is relatively different from all cases.

The prevalence of hyperparathyroidism is slightly higher in patients with renal involvement (reported in our studies with 90% prevalence) than previous comprehensive report (84.2%).

A screening for hypertension, UTI, microalbuminuria, metabolic acidosis, nephrocalcinosis, nephrolithiasis, and other renal function (particularly tubular) indicators is recommended to be considered in the examination of APECED patients (Table 2).

**Table 2:** Suggested screening test to detect renal involvement in APECED patients. This suggestion should be further completed by other clinicians and studies

Renal disorder	Prevalence in this study	Recommended screening for early detection of diseases
Nephrocalcinosis	52%	Ultrasonography, Urine Analysis
TIN	32%	Urine Analysis, Renal biomarkers
Hypertension	18%	Blood pressure screening during each visit
Glomerulonephritis	4%	Urine Analysis

## Limitation

This study has also some limitations. Since APECED is relatively a rare disorder, the number of included patients in studies was limited, and most of the included studies were case reports. The results of this study should be confirmed by future studies, especially cohorts and case controls which could enhance our understanding and knowledge of this disease. Furthermore, since APECED is a rare disorder, most of the included studies were case series, case reports studies. Therefore, we were unable to prefom an appropriate and solid statical analysis. There is a lack of investigations, especially cohort studies on APECED and renal involvement. Future studies, particularly cohort studies is warranted to update our understanding regarding this rare condition.

## Conclusion

Taken together, renal complications in APECED represent a significant issue that should be monitored and considered in managing these patients to preserve renal function and improve patients’ outcomes. This is the first study reviewing renal involvement in patients with APECED. The results of this study could enhance our understanding of the impact of this disease on patients.

## Supplementary Information

Additional file 1.

## Data Availability

The datasets used and/or analysed during the current study are available from the corresponding author on reasonable request.
